# Early initiation of therapy with vosoritide: case report. Description of the first Italian patient affected by achondroplasia treated with vosoritide before 2 years of age

**DOI:** 10.3389/fped.2026.1741018

**Published:** 2026-07-06

**Authors:** Mila Ann Kalapurackal, Petra Wanker

**Affiliations:** 1 Department of Pediatrics, Hospital of Bolzano (SABES-ASDAA), Teaching Hospital of Paracelsus Medical University (PMU), Bolzano, Italy; 2 Department of Neonatology, Hospital of Bolzano (SABES-ASDAA), Teaching Hospital of Paracelsus Medical University (PMU), Bolzano, Italy

**Keywords:** achondroplasia, case report, *FGFR3* gene, infant, skeletal dysplasia, vosoritide

## Abstract

Achondroplasia, the most common skeletal dysplasia, is mainly characterized by disproportionate short stature. Affected patients present a reduced linear growth, and the greatest decrease in growth velocity is evident in infancy. Vosoritide is the first approved drug to improve linear growth in children affected by achondroplasia. Since 2023, it is approved in children from birth by the FDA, and from 4 months of age by the EMA. However, until October 2025, the prescription and reimbursement of vosoritide in Italy was only possible starting from 2 years of age. We present the case of the first patient affected by achondroplasia treated with vosoritide in Italy before 2 years of age, managed thanks to the approval by the local rare diseases commission. Therapy with vosoritide was initiated at 7 months of age. The infant presented no severe adverse effects. She completed 12 months of therapy, at which time an improvement of +0.35 SD in length, according to growth curves specific for girls affected by achondroplasia, was documented. This result is in line with previous studies and underlines the importance of an early therapy initiation.

## 1 Introduction

Achondroplasia is the most common form of skeletal dysplasia, with an incidence of approximately 1 in 25,000 births worldwide, with no recognized prevalence in sex or race (1, 2). It is caused by an autosomal dominant gain-of-function mutation in the gene coding for the Fibroblast Growth Factor Receptor 3 (FGFR3), which determines an activation of a signalling pathway that results in impaired endochondral ossification (3, 4). The clinical manifestations include macrocephaly, midface hypoplasia and rhizomelic disproportionate short stature, leading to a final adult height of approximately 132 cm in males and 124 cm in females (5). Linear growth deficit is particularly evident in infancy, with a rapid decrease in growth velocity in children with achondroplasia in the first two years of age compared with the non-affected population (5).

Vosoritide, a recombinant C-type natriuretic peptide analogue, is the first approved drug to improve linear growth in patients affected by achondroplasia (6). It was approved by the European Medicines Agency (EMA) and the US Food and Drug Administration (FDA) in 2021 for children aged 5 years and older with genetically confirmed achondroplasia whose epiphyses are not closed. In 2023, the approval was extended to infants and children aged 4 months and older by the EMA (7) and from birth by the FDA (8).

As of today, the prescription of vosoritide in Italy is reimbursed only through an Italian Medicines Agency (AIFA) registry. Its reimbursement was limited to children over 2 years of age until the end of October 2025, by when it was extended to infants from 4 months of age, in accordance to EMA indications (9).

We present the case of the first infant affected by achondroplasia treated with vosoritide under 2 years of age in Italy, managed thanks to the approval by the Commission for Rare Diseases of the Autonomous Province of Bolzano.

## 2 Case description

A female newborn was delivered with Cesarean section at 36 + 4 weeks of gestation. A skeletal dysplasia was suspected during the 20th week of gestation, due to shortness of the femur (5th percentile, head circumference 50th percentile), with progressive worsening at subsequent ultrasounds. An amniocentesis was performed at 31 weeks of gestation which documented a *de novo* heterozygous mutation of the *FGFR3* gene (c.1138G>A). The diagnosis was confirmed at birth due to typical clinical manifestations (rhizomelia, relative macrocephaly, midface hypoplasia) and genetic testing on the newborn. At birth she presented with 1- and 5-min Apgar scores of 8 and 9, respectively, weight = 2,140 g (−1.28 Standard Deviation Score—SDS), length = 42 cm (−2.19 SDS), and head circumference = 33 cm (0.23 SDS). Respiratory support with Continuous Positive Airway Pressure—CPAP was administered for the first two days of life due to initial respiratory distress. She presented no other perinatal difficulties. Table 1 reports the timeline regarding the patient's clinical course.

**Table 1:** Timeline about the clinical course.

Life period	Signs, symptoms, diagnostic tests and clinical interventions	Biometric data
20th week of gestation	Suspicion for skeletal dysplasia due to shortness of the femur (5th percentile)	
36th + 4 weeks of gestation	Confirmation of skeletal dysplasia at birth due to: typical clinical manifestations (rhizomelia, relative macrocephaly, midface hypoplasia); genetic confirmation of a *de novo* heterozygous mutation of the *FGFR3* gene (c.1138G>A)	Weight = 2,140 g (−1.28 SDS) Length = 42 cm (−2.19 SDS) Head circumference = 33 cm (0.23 SDS)
First 2 days of life	CPAP due to initial respiratory distress	
2 weeks	MRI: foramen magnum stenosis with no signs of medullary compression	
3 months	Hospital admission due to transient reduced weight gain	
5 months	Polysomnography: rare brief central apneas, with no severe desaturations (AHI = 3.8/h).	
7 months	Normal heart ultrasound and blood tests Vosoritide initiation	Weight = 5.2 kg (−1.36 SDS according to growth charts of girls affected by achondroplasia) Length = 56.6 cm (−0.87 SDS); Head circumference = 44 cm (−1.10 SDS) Arm span = 49.5 cm
11 months	Achievement of autonomous sitting	
12 months	MRI: confirmation of foramen magnum stenosis with no signs of medullary compression	
18 months	Polysomnography: negative	
19 months	Achievement of independent walking	Weight = 7.15 kg (−1.87 SDS) Length = 67.2 cm (−0.52 SDS) Head circumference = 49.5 cm (−1.10 SDS) Arm span = 60 cm

AHI, Apnea–hypopnea index; CPAP, continuous positive airway pressure; MRI, magnetic resonance imaging; SDS, standard deviation score.

## 3 Diagnostic assessment

Due to slight hypotonia and as per international consensus regarding patients with achondroplasia, she underwent a cerebral Magnetic Resonance Imaging—MRI at 2 weeks of age, which documented a foramen magnum stenosis with no signs of medullary compression (10).

She was admitted to the hospital at 3 months of age because of a transient reduced weight gain. All tests performed resulted negative, and the infant improved with simple positional remedies in the following weeks.

She underwent polysomnography at 5 months of age which documented rare brief central apneas, with no severe desaturations (Apnea–Hypopnea Index—AHI = 3.8/h).

At 7 months of age, the Commission for Rare Diseases of the Autonomous Province of Bolzano granted the possibility of initiating a treatment with vosoritide. Before initiation, a heart ultrasound and blood tests (cell blood count, hepatic, renal and thyroid function, albumin, glycemia) were performed and resulted normal. Vosoritide was administered at 30 µg/kg/day, as per international guidelines. At therapy initiation the infant presented with a weight of 5.2 kg (−1.36 SDS), length = 56.6 cm (−0.87 SDS), head circumference = 44 cm (−1.10 SDS), and arm span = 49.5 cm. All SDS scores refer to growth charts of girls affected by achondroplasia (5).

The therapy was regularly administered from the parents, well tolerated, and no adverse effects were reported.

Regular evaluations and measurements were performed in a clinical setting every 3 months and showed a slight but progressive increase in length percentiles in growth curves specific for female patients affected by achondroplasia.

An MRI was performed at 1 year of age and confirmed the foramen magnum stenosis without any medullary involvement (Figure 1). The last polysomnography, performed at 18 months of age, resulted negative. These examinations were repeated because the initial evaluations showed mild abnormalities.

**Figure 1:**
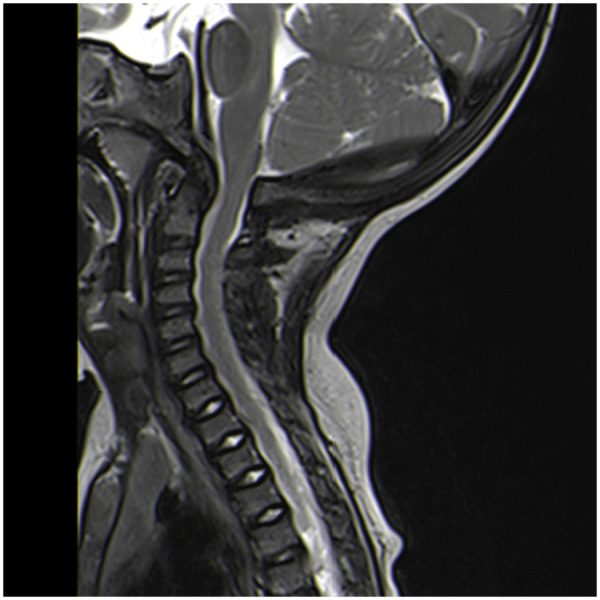
MRI image (T2 TSE sagittal view) performed at 1 year of age documenting the slight foramen magnum stenosis without medullar involvement.

The infant showed normal neurological development, achieving independent ambulation at 19 months of age.

The last evaluation was undertaken at 19 months of age and after 12 months of therapy with vosoritide: the infant weighed 7.15 kg (−1.87 SDS), was 67.2 cm (−0.52 SDS) long, her head circumference was 49.5 cm (−1.10 SDS), and her arm span was 60 cm. Her growth velocity was 11.4 cm/year, calculated during a 6-month period.

## 4 Discussion

Achondroplasia is characterized by a disproportionate short stature due to a disruption of endochondral growth of the long bones. In comparison to the healthy population, children with achondroplasia experience reduced linear growth, particularly during the first 2 years of age, when these infants grow approximately 15 cm less than non-affected children. More specifically, 33% total height deficit in adulthood occurs during the first 2 years of age in boys and 37% in girls affected by achondroplasia (5).

While several drugs are being studied in patients with achondroplasia with promising results, as of today vosoritide is the only approved drug to improve growth of long bones in these children. It has recently been approved for administration in infants starting from 4 months of age by EMA (7). However, until October 2025, its use in Italy has been limited to children aged 2 years and older through AIFA registry. At the end of October 2025, AIFA approved the reimbursement of vosoritide starting from 4 months of age, in accordance to EMA indications (9).

The patient presented in this case report managed to initiate therapy with vosoritide early, at 7 months of age, thanks to the approval of the local rare diseases commission. We documented a 0.35 SDS improvement in the infant's length in growth curves of girls affected by achondroplasia after one year of therapy (5). This improvement is comparable to that documented by other authors, who reported a mean of 0.25 (−0.02 to 0.53) gain in length *Z* scores at 52 weeks of therapy in infants aged 3 months and older (8). According to the Summary of Product Characteristics, the most common adverse events associated with vosoritide administration include hypotension, vomiting, injection-site reactions, and increased blood alkaline phosphatase levels; none of these adverse events were observed in our patient during the follow-up period (11).

This case report highlights the importance of an early initiation of therapy in infants with achondroplasia, in order to contrast the severe growth deficit which occurs especially during the first 2 years of age and thus improve their final height in adulthood.

## Data Availability

The original contributions presented in the study are included in the article, further inquiries can be directed to the corresponding author/s.
